# Closing the Gaps on Medical Education in Low-Income Countries Through Information & Communication Technologies: The Mozambique Experience

**DOI:** 10.26717/bjstr.2019.16.002875

**Published:** 2019-03-29

**Authors:** Ben Lauro Zavale, Eliah Aronoff Spencer, Carvalho Guilundo, Robert Schooley, Sam Patel, Emília Virgínia Noormahomed, Ana Olga Mocumbi

**Affiliations:** 1 Hospital Maputo Central, Mozambique; 2 University California San Diego, United States; 3 Mozambique Institute for Health and Research (MIHER/MEPI), Mozambique; 4 Faculty of Medicine, Universidade Eduardo Mondlane, Mozambique; 5 Instituto Nacional de Saúde, Mozambique

**Keywords:** Transformative Medical Education, Information & Communication Technologies, M-Health, Distance Learning

## Abstract

**Background:**

Medical training in developing countries has continuously faced challenges to produce the needed number of cadres and maintain the needed quality standards. Mozambique, a low-income country in Southern Africa, has a major disparity in distribution of medical doctors in the country and only a small proportion are trained as specialists and/or retained as faculty. In this context, we thought that Information and Communication Technologies (ICT) are an attractive tool to support expansion of medical training and residency programs and designed a strategy for its use to promote changes in the learning environment of a university teaching hospital in this country.

**Approach:**

Beginning in 2010-under the Medical Education Partnership Initiative between Universidad Eduardo Mondlane, Mozambique, and University of California San Diego, United States of America - we conducted extensive interviews to 21 UEM faculty and medical trainees to assess barriers to education and medical care at the major referral hospital in Mozambique. Then, changes were made to address the issues raised mainly through building an ICT infrastructure to improve connectivity, improving access to medical information, distributing communication and mobile medical devices, as well as fostering exchange between students, residents and faculty members. These changes were tracked years after to evaluate adoption.

**Main Findings & Discussion:**

Internet access with large bandwidth and devices such as tablets and computers were distributed to increase access to medical information. The students: resident ratio improved from 13:1 to 5:1 at the end of the project. Additional 25 new faculty members were involved in clinical training, mainly through incentives such as faculty development courses and research training. Teleconferences and other exchanges using ICT have evolved from being used as a platform for weekly clinical rounds and case discussions, to become a day-to-day tool for implementation of quality improvement processes and research projects. New exchange programs between local and foreign institutions were fostered to create a growing network with over 20 institutions at the end of the program. Importantly, these changes persisted beyond the project, and constituted a driver for transformative education and distance learning.

**Conclusion:**

Context-tailored use of ICT and mobile medical devices transformed medical education by improving the learning environment, addressing scarcity and low quality of trained doctors in a low-income setting of Africa. This strategy has the potential to reduce health disparities and contribute to achieving universal health coverage. Efforts to guaranty sustainability and health professional’s retention are warranted.

## Introduction

Medical training in developing countries has continuously encountered challenges to sustain quality and to produce the needed number of cadres. Issues related to healthcare systems and incapacity to retain trained workforce, as well as to inadequacy of the training environment, are among the causes of low quality of medical training and the reduced number of specialists in low-resourced areas in Africa [1]. This continent has the lowest percentage of healthcare workforce worldwide (3%) and the lowest relative health expenditure, in clear contrast with its 24% of global disease burden that it hosts [2]. Mozambique, a low-income country in Southern Africa, ranks 181st among all nations in annual per capita healthcare expenditures (<$50), and 195th in health systems, with less than 1000 doctors for a population of 23 Million in 2013 [3]. In the beginning of this century more than half of the country’s 712 doctors worked in Maputo, with an estimated doctor to patient ratio of 1:4,000 compared to 1:60,000 in the North. Low yearly output of medical graduates, insufficient faculty at medical schools and inadequate salaries to retain the most skilled in training institutions all contribute to the slow increase in numbers of trained faculty in teaching hospitals. Thus, like in many other parts of the African continent, [4–6] only a small proportion of medical doctors are trained as specialists [7].

The importance of Information Communication Technologies. (ICTs) for developing countries is still the subject of strong debates. The government of Mozambique has developed an ICT policy since mid-90’s, with education, health and human resources development being the most important priority areas [8]. However, a study in three provinces identified lack of ICT-skills and education as well as poorly developed infrastructure and networks of support, as major challenges [9]. A general recommendation from this study was the need for training of health workers and managers, as well as the design of master’s and PhD programmed in ICT and health information systems. Recognizing the need to strengthen medical education to improve its health education institutions the Medical Education Partnership Initiative (MEPI) in Mozambique - Universidade Eduardo Mondlane (UEM) in Mozambique, in collaboration with the University of California, San Diego (UCSD) - prioritized strengthening of its medical training system, including the residency programs. We hypothesized that to build local capacity for medical education, there was need to increase access to Information and Communications Technology (ICT) aiming at increasing the number and quality of medical graduates as well as at delivering better patient care, implement quality improvement processes and incorporate research into clinical practice.

## Approach

### Situational Analysis (Observations, Theme Selection)

**Figure 1:**
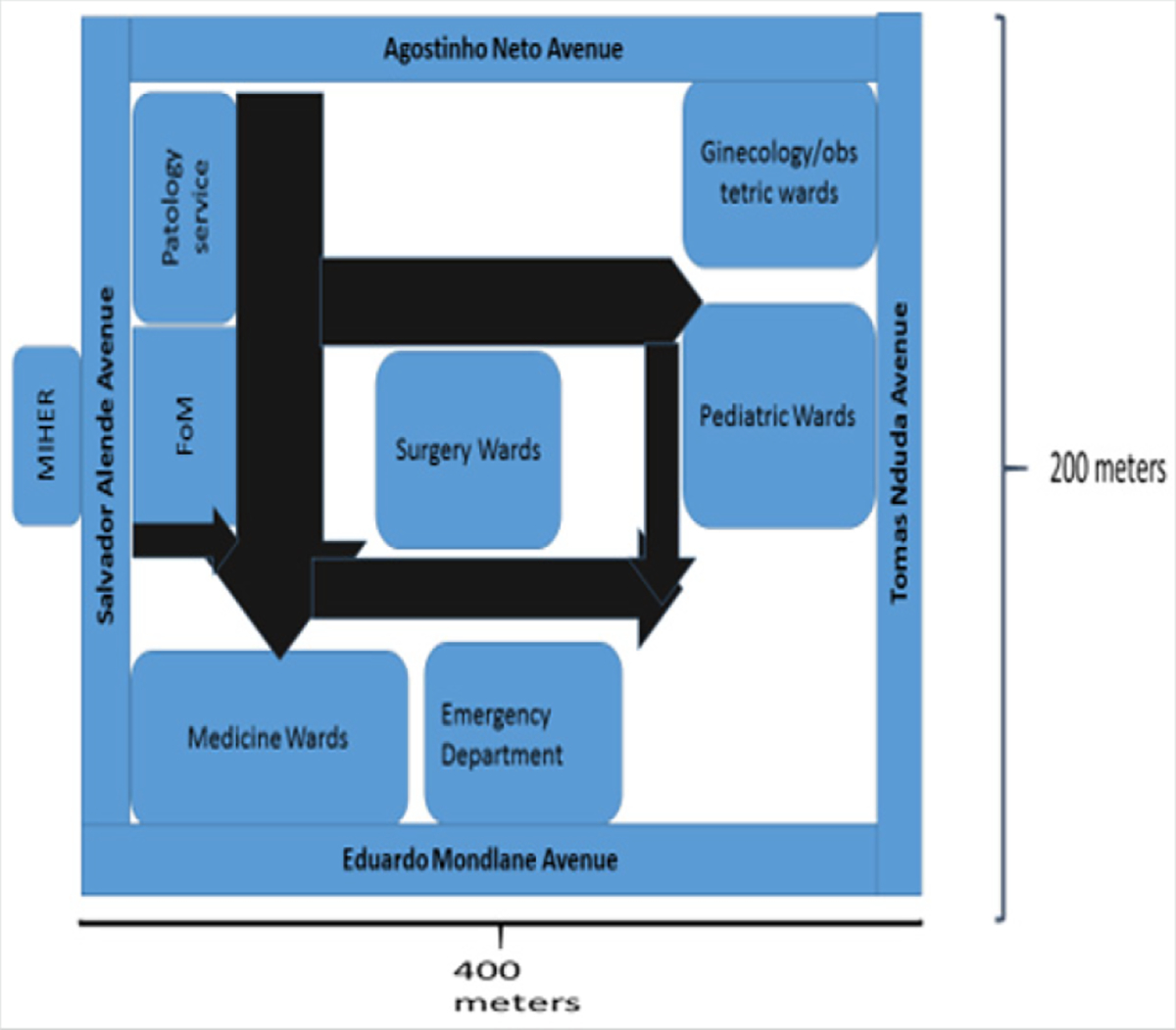
Diagram of the FoM/MCH complex including the location of the main departments served by the library and research support center (MIHER).

At the outset of its partnership with the University of California, San Diego (UCSD) in 2008, UEM was one of only 2 medical schools graduating MD in Mozambique. In addition, two public Universities had been founded since 2006: UniLurio in north of Mozambique and UniZambeze in Central Mozambique, graduated their first classes in 2014 (graduated 47 MD) and 2015 (graduated 28 MD) respectively. Our study is based on MCH, the teaching hospital for UEM, which was graduating per year around 40 to 50 students. Specializations in medical areas was affiliated with MoH and located exclusively at Maputo Central Hospital (MCH), a 1200 bed facility working as the main referral health for specialized care in the country. MCH is adjacent to the UEM Faculty of Medicine (FoM) (Figure 1) and is also the primary teaching facility for other medical related professions (nursing school and health technicians). Didactic technologies were restricted to data projection; distance learning and online courses were non-existent, while library resources for students and residents were paper-based. A 24 hours’ library service to medical students proved non feasible at that time.

Textbooks for checkout were scarce, and thus they were heavily controlled with students having only access to them indoors. At FoM a single computer workroom was available for the entire student population (in 2010 there were 959 students) with minimal Internet accessibility provided through a local company. At MCH library resources consisted of old textbooks and only ad hoc use of computers occurred [10]. Beginning in 2010 we conducted extensive interviews to 21 UEM faculty and medical trainees to assess barriers to education and medical care and identify priorities for interventions; this included availability of resources, IT needs, as well as barriers to communication and learning [7]. Clear themes for effective change emerged from initial observations and surveys. These included the need for improved:

- Access to accurate and recent clinical knowledge directed to medical education and care.;
- Access to patient data including demographics, laboratory data, radiology and outside health records;
- Quantity and quality of teaching both in classroom (or case conference) as well as at the bedside;
- Communication with colleagues and experts for the purpose of daily patient care as well as quality improvement and clinical research projects.

These themes mapped key aspects of daily clinical workflow by the for local staff including improved access to electronic clinical references and support tools; data/information support tools, eg patient registration, demographics, medical history and laboratory findings; new approaches (integrating best practices and technology) to education, research and care capacity building; better communication technologies for team and expert coordination. The assessment allowed the team to determine and recommend interventions which might improve medical training, using ICT.

### Phase 1: Setting an Infrastructure

We conceptualized a basic ICT framework comprised of broadband Internet, low cost tablets, work-stations and open source or freemium software services. These elements were used to generate a technical roadmap for sustainable development and implementation. In general, any solution was screened for four basic attributes: affordability, quality, interoperability and usability. We installed local intranet with backup power and server using readily available cabling, switches, and backup power supplies in Mozambique (supplemental). All installation and service were carried out by UEM Center for Informatics engineers with engagement of MEPI collaborators. Broadband Internet for the Central Hospital and Faculty of Medicine was transmitted via line-of-sight microwave tower from the UEM SEACOM link (a fiber optic cable along Africa’s east coast) that was established in partnership with the MEP. Wireless connections were installed at common halls, library and primary teaching facilities, all internal medicine wards, surgical and specialty wards, gynecology, pediatrics, emergency room, pathology, radiology and laboratory facilities.

A central server for secure intranet and Internet portal was installed in the Medical School central library.48 tablets and 60 Computer libraries were installed at the Medical School and Central Hospital Libraries and teaching facilities were fitted with audiovisual equipment for teleconferencing and distance learning. Teleconferencing equipment included Logitech and Sony web cameras Logitech Bluetooth microphone with feedback control and muting, speakers and video projectors. Teleconferencing was carried out using standard applications such as Goto webinar, Skype or Google hangouts. At the present, no secure conferencing application has yet been adopted and thus teleconsultation involving private health information has yet to be adopted. A web portal with single sign on was created to unify technologies used in the MEPI (moz.digitalhealthspace.org). Open source tools for sharing and discussing clinical cases (dokbot.org), differential diagnosis (doknosis.org) and data collection, sharing and visualization (getkeep.org, keep. Health iOS app store) were developed and tested in Mozambique [7,11,12].

### Phase 2: Fostering Transformative Learning

Availability of Internet was combined with new and affordable technologies such as tablets, ultra-low-cost desktops and open source software to craft a cohesive yet affordable solution to the MEPI aims. Furthermore, basic tools that could be used for distance learning, were introduced, namely PC-based programs for ECG and portable ultrasound machines. In general, the implementation plan consisted of “small pilot studies” that were expanded or amended based on rapid feedback and flexibility of the teams and technology. Each pilot study was conducted to test and evaluate solutions to problems detected during the HCD Process. Initial technology pilot studies included:

- weekly clinical case conferences between UEM/UCSD (there have now been >200 of these teleconferences since 2009).
- interned patient Bedside teaching using tablets and online and offline resources.
- Clinical research pilots using customized mobile data collection applications on smart phones.
- Use of portable ultrasound in medical training, as an extension of physical examination.

The first of these clinical research pilots, centered on improvement of blood culture utilization and reporting has recently been reported. This experience was also used to support the growth of UniLurio University through linking/connecting them to UEM, and to support two master’s degree programs. Finally, portable ultrasound machines were provided to the IM Department for use in training the next generation of faculty members; young specialists and residents were trained for screening and abbreviated ultrasound evaluation of the most common conditions seen in this setting. All these programs use distance learning and innovative approaches to take advantage of off-site faculty members for teaching and mentoring/tutoring students. This was also done to allow decentralization of specialized care and diagnostic capacity, since the residents are expected to be working in remote areas in the near future.

### Phase 3: Tracking Changes

To evaluate the changes beyond the project we used the tracking system implemented at the Universities’ research support center (MIHER), reviewed annual reports, visited MCH and established contact with the former residents.

## Main Findings

Before the implementation of our project internet at MCH was accessible less than 1% of the time and the bandwidth was 100kB/second; after intervention it substantially improved with accessibility currently being more than 90% of the time with bandwidth increased to 250kB/second. Despite occasional slow download speeds that require backup cellular-internet, the number of teleconferences in MCH still increased per year. In addition, in contrast with the inexistence of any tablet at the beginning of the project there were 48 at its end; similarly, the number of computers used in the Department increased from 4 to 60 at the end of the intervention. To date most students on clinical training have their own device. The students per resident ratio before the project had started was 13:1, decreased to 5.6:1 at the end of the program and has remained at 5.4:1 up to now. Similarly, the number of new faculty members involved on clinical training of students and residents increased from 5 at the beginning of the program to 14 between 2012–2014, and 25 between 2015–2018. Teleconferences and other exchanges using ICT have evolved from being used as a platform for clinical rounds and case discussions, to become mostly used for design and implementation of quality improvement processes and research projects (Table 1).

When the project started three junior specialists and seven senior residents were selected to be trained to support the five specialists who constituted the faculty members dedicated to clinical training of medical students and residents. These selected professionals benefited from a career development program which included research training and introduction to health professional’s education. To date all remain connected to clinical training in several public and private institutions: three are currently heads of the medical departments in their institutions, leading programs that use e-devices for teaching. Of the 7 residents who became specialists, one obtained master’s degree and is currently completing a PhD program and two are currently ending their MSc programs. (Table 2) Before the start of the project UEM-Faculty of Medicine and MCH were only connected to Barcelona University (Spain) for joint clinical rounds and discussions twice a month. As mentioned above, teleconferences and other exchanges using ICT were started to support clinical rounds and case discussions, progressively evolved to include new partners and institutions, and they now involve not only other institutions in North America, Africa, Europe, India and Latin America.

This network is now used not only for improving medical training but also for different quality improvement and clinical research projects, including currently running research projects with over 25 institutions. Additionally, the research projects expanded to others Mozambican universities as well as to Africa, European and American Universities, allowing in exchange between residents and mentors from local institutions and their partners from abroad.

## Discussion

We have shown progressive improvement of learning environment in a referral teaching hospital from a low-resourced setting as a result of investments in human resources training, infrastructure improvement and free access to medical literature for students, residents and faculty members. This strategy was used to facilitate point-of-care access to the medical literature and enhance health information technology skills to all health professionals. Additionally, Internet has allowed distance learning (courses, teleconferences, telemedicine sessions), and open source software has facilitated data collection and high-quality research in this teaching hospital. Importantly, computer libraries remain open and are fully utilized to date. Moreover, currently mobile PC-based or POC equipment are routinely used in health professional’s training and research. Our initial observations quickly focused our work on the creation of opportunities for local students and doctors to take advantage of the global technology revolution through the use of smart phones, tablets, and open-source computing. These technologies have become widespread in developing nations, and platforms based on these technologies show promise for supporting even the most demanding healthcare systems [13,14–23].

However, the healthcare and education sectors in poor countries such as Mozambique have lagged in adopting and implementing these transformative technologies [22, 24–27]. However, ICT and m-health devices should not be considered unaffordable or inappropriate in low-income settings; they may rather have great potential to change the way medical education is currently delivered and may contribute to improve quality of care and reduce health disparities. Based on early successes as well as program evolution, use of these technologies has become regular in MCH and the UEM Medical School, and has catalyzed a gradual transformation of medical education. For example, “journal club” in which recent medical literature is discussed, is now a regular feature of post-graduate medical education, whereas five years ago, residents had virtually no access to this literature, or a forum in which to discuss it. Several successes can be highlighted after implementation of this project. Internet has become the primary means for communication (email), study (eLibrary, search), distance learning (teleconferences, Masters courses lectures, ultrasound training) and collaborative research (mentorship of local health professionals by partners in external academic institutions).

Importantly, e-devices are now acquired without any costs to the training institution or health provider (either personal computers or android devices are being). The medical community including faculty members and residents are currently connected through www.medicinainternamz group and MCH - Plataforma Clínica, which enable them to discuss patients’ issues or share literature of clinical interest. Two links, using text messaging platform (WhatsApp group), have also been created: one allows communication between the IM Department and the Laboratory Services to report results from critically ill patients to their assisting doctors, while the second connects the Emergency Medicine room at the teaching hospital to Intermediate Care Services at peripheral hospitals referring to it, thus contributing to readiness to act when transferring critical patients to MCH. At inception of the program increasing the number of postgraduate trainees was defined as a major MEPI goal [7]. Recognizing this opportunity, the Ministry of Health chose to more than double the size of Mozambique’s residency training programs; this resulted in an increase from 23 residents between 2009 and 2011 to three times more in the next three years. O the hand, from 5 faculty members attached to the wards for residence programs in 2011 we now have 24 (Table 1).

These changes contributed largely to the improvement in the learning environment, and the number of teleconferences and other student-residents-faculty exchanges, allowing daily journal clubs and scientific sessions to become a regular feature of post-graduate medical education. MEPI program in Mozambique focused strongly on strengthening institutional capacity in order to sustain longer-term progress, and therefore supported specific faculty development programs, particularly for clinical training. The project was a learning process with several challenges that need to be considered. Training activities were frequently hampered by high burden of care provision on highly trained professionals. On the other hand, the challenges faced by the healthcare systems to sustain quality of care in teaching health facilities pose an import barrier to good quality of training in such settings. Following this experience research has become an essential part of our residency training. However, efforts should be made to sustain these changes and find incentives to retain the newly trained specialists who became involved in training.

## Conclusion

Context-tailored use of information and communication technologies - including m-devices - is an approach that can be used to deliver better medical education and address scarcity of trained human resources, contributing to reduction of health disparity and to achieve universal health coverage in low-income settings. Further research on strategies to sustain these successes is warranted.

## Figures and Tables

**Table 1:** Distribution of informatics equipment obtained for e-learning activities, type of training activities implemented and number of beneficiaries before, during and after project implementation.

E-learning equipment & activities	2009 – 2011	2012 – 2014	2015 – 2018
Internet/Bandwidth	<1%/100 kB/s	~50%/50MB/s	>90%/250MB/s
Tablets	0	48	250*
Computers	4	60	Own computers
Teleconferences/year	40	52	64**
Ultrasound courses with distance learning	0	4	3
Students***/Residents	301/23	381/68	274/51
Faculty members involved	5	14 new Faculty	25 new Faculty
Collaborating Institutions (research projects)	5	12	24

* most students have their own devices

** used not only for clinical rounds and case discussions, but also for research projects

*** students which were on clinical training

**Table 2:** Career progression and use of e-devices in training for the seven junior specialists (JS) and senior residents (SR) that constituted the cohort of 2011, when the project started.

Qualifications	Post-Graduate Studies	Current Position in teaching	Use of e- devices
JS1	IM JS; Public Teaching Hospital	Head of Department; Faculty at UEM	Yes PU; SK; TC; EM
JS2	IM JS; Private Non-profit Hospital	Head of Department; Supports residency program	Yes PU; SK; EM
JS3	IM Specialist Physician; Private Hospital	None	Yes EM
SR1	IM JS; Public Teaching Hospital University Assistant	MSc; PhD student Faculty at UEM	Yes PU; SK; TC; EM
SR2	IM JS; Public Teaching Hospital	MSc Student; Faculty at UEM	Yes PU; SK; TC; EM
SR3	IM JS; Public Teaching Hospital	University	Yes PU; SK; TC; EM
SR4	IM JS; Public Teaching Hospital	Faculty of Medicine	Yes PU; TC; EM
SR5	IM JS; Private Non-Profit Hospital	Supports practical tutorials on residency program	Yes PU; SK; TC; EM
R6	IM Specialist Physician; Non-Governmental Organization	International Training & Education Center for Health	Yes EM; VL
R7	IM JS; Public Teaching Hospital	Head IM Department; Faculty at UNILURIO	Yes EM; VL

EM email; JS junior specialists; PU portable ultrasound; SK skype; TC web-based teleconferences VL virtual library; UEM Universidade Eduardo Mondlane; UNILURIO Universidade Lúrio.
